# Maximum fluorescence and electron transport kinetics determined by light-induced fluorescence transients (LIFT) for photosynthesis phenotyping

**DOI:** 10.1007/s11120-018-0594-9

**Published:** 2018-10-24

**Authors:** Beat Keller, Imre Vass, Shizue Matsubara, Kenny Paul, Christoph Jedmowski, Roland Pieruschka, Ladislav Nedbal, Uwe Rascher, Onno Muller

**Affiliations:** 1 Institute of Bio- and Geosciences, IBG-2: Plant Sciences, Forschungszentrum Jülich GmbH, 52425 Jülich, Germany; 2 Institute of Plant Biology, Biological Research Center, 6726 Szeged, Hungary; 3 Present Address: Molecular Plant Breeding, ETH Zürich, 8092 Zurich, Switzerland

**Keywords:** Fluorescence transient, Photosynthesis, Fast repetition rate, Electron transport kinetics

## Abstract

Photosynthetic phenotyping requires quick characterization of dynamic traits when measuring large plant numbers in a fluctuating environment. Here, we evaluated the light-induced fluorescence transient (LIFT) method for its capacity to yield rapidly fluorometric parameters from 0.6 m distance. The close approximation of LIFT to conventional chlorophyll fluorescence (ChlF) parameters is shown under controlled conditions in spinach leaves and isolated thylakoids when electron transport was impaired by anoxic conditions or chemical inhibitors. The ChlF rise from minimum fluorescence (*F*_o_) to maximum fluorescence induced by fast repetition rate (*F*_m−FRR_) flashes was dominated by reduction of the primary electron acceptor in photosystem II (Q_A_). The subsequent reoxidation of Q_A_^−^ was quantified using the relaxation of ChlF in 0.65 ms (*F*_r1_) and 120 ms (*F*_r2_) phases. Reoxidation efficiency of Q_A_^−^ (*F*_r1_/*F*_v_, where *F*_v_ = *F*_m−FRR_ − *F*_o_) decreased when electron transport was impaired, while quantum efficiency of photosystem II (*F*_v_/*F*_m_) showed often no significant effect. ChlF relaxations of the LIFT were similar to an independent other method. Under increasing light intensities, *F*_r2_′/*F*_q_′ (where *F*_r2_′ and *F*_q_′ represent *F*_r2_ and *F*_v_ in the light-adapted state, respectively) was hardly affected, whereas the operating efficiency of photosystem II (*F*_q_′/*F*_m_′) decreased due to non-photochemical quenching. *F*_m−FRR_ was significantly lower than the ChlF maximum induced by multiple turnover (*F*_m−MT_) flashes. However, the resulting *F*_v_/*F*_m_ and *F*_q_′/*F*_m_′ from both flashes were highly correlated. The LIFT method complements *F*_v_/*F*_m_ with information about efficiency of electron transport. Measurements in situ and from a distance facilitate application in high-throughput and automated phenotyping.

**Electronic supplementary material:**

The online version of this article (10.1007/s11120-018-0594-9) contains supplementary material, which is available to authorized users.

## Introduction

Photosynthetic processes, from light absorption by the chlorophyll-based pigments through charge separation in the photosystem II (PSII) reaction centers and sequential electron transport, are related to the redox state of the primary quinone electron acceptor (Q_A_) and coupled to the signature of chlorophyll fluorescence (ChlF) (Kautsky and Hirsch 1931; Baker 2008; Müh et al. 2012). Based on ChlF, parameters such as the maximum quantum efficiency of PSII (*F*_v_/*F*_m_) and non-photochemical quenching (NPQ) estimating the proportion of absorbed light energy utilized for PSII photochemistry and non-photochemical energy dissipation, respectively, were established (Butler 1978; Baker 2008; Lazár 2013). The quick assessment of ChlF makes this signal a powerful tool for plant phenotyping (Furbank and Tester 2011; Fiorani and Schurr 2013). Phenotyping requires characterization of a large plant set which needs to be completed before significant changes in the measured traits occur. This is particularly difficult when phenotyping photosynthesis because this process is highly dynamic and sensitive to environmental conditions (Ananyev et al. 2005a; Kono and Terashima 2014).

In order to determine *F*_v_/*F*_m_, the ChlF signal is compared under conditions when Q_A_ is in a fully oxidized state resulting in minimal ChlF (*F*_o_) respective to the fully reduced state resulting in maximal ChlF (*F*_m_). Two approaches using strong light pulses are widely accepted to reduce Q_A_ fully: the single turnover flash (STF) and the multiple turnover flash (MTF) (Kalaji et al. 2017). A saturating STF has to provide high enough excitation power to induce one single charge separation in all PSII reaction centers and fully reduce Q_A_ in order to yield maximum ChlF level (*F*_m−ST_) (Malkin and Kok 1966; Schreiber 1986a; Samson and Bruce 1996; Kolber et al. 1998; Steffen et al. 2001). The excitation flash needs to be short enough (from fs to few µs) to prevent reoxidation of Q_A_^−^ and reexcitation of PSII reaction centers (Malkin and Kok 1966; Belyaeva et al. 2014). In contrast, a saturating MTF requires at least 0.2-s duration of excitation at a few 1000 µmol photons m^−2^ s^−1^ (Ögren and Baker 1985; Schreiber et al. 1986b; Schreiber 2004). Within this time range, Q_A_ is reduced and reoxidized several times followed by reduction of the plastoquinone (PQ) pool and electron transfer to Photosystem I (PSI) (Vernotte et al. 1979; Schansker et al. 2005). MTFs ultimately result in an about 50% higher maximum ChlF level (*F*_m−MT_) compared to *F*_m−ST_ (Schreiber 1986a; Schreiber et al. 1986b; Schansker et al. 2011). The difference between *F*_m−ST_ and *F*_m−MT_ was named the thermal phase because it is dependent on temperature, i.e., it is rate-limited (Delosme 1967). Later, the ChlF rise of the thermal phase was related to electron transport kinetics, particularly the accumulation of secondary quinone acceptors (Q_B_) in a reduced state (Strasser et al. 1995; Lazár 2006). However, the origin of the thermal phase is not yet localized due to the complex and overlying kinetics of different electron transport processes (Rascher and Nedbal 2006; Müh et al. 2012) and the probable involvement of additional ChlF quenchers (Schansker et al. 2011, 2014; Prášil et al. 2018; Magyar et al. 2018).

Alternatively, electron transport kinetics in the dark-adapted state were studied by following reoxidation of Q_A_^−^ coupled to ChlF relaxation after a STF (Vass et al. 1999; Petrouleas and Crofts 2005). According to an exponential decay model with three time constants, ChlF relaxes due to electron transport from Q_A_^−^ to Q_B_ with a time constant (*τ*_1_) of 0.1–0.2 ms when the Q_B_ site is occupied by a PQ (Bowes and Crofts 1980; Vass et al. 1999; Shinkarev 2004; Petrouleas and Crofts 2005). The second exponential component represents the reoxidation of Q_A_^−^ which had initially no PQ molecule bound (Taoka and Crofts 1990; Petrouleas and Crofts 2005). Therefore, this time constant (*τ*_2_) represents the binding of PQ molecule to the Q_B_ site of PSII and is estimated to be between 2.2 and 10 ms (Vass et al. 1999; Eshaghi et al. 2000). The third component (*τ*_3_) is slow (500 ms to seconds) and interpreted as a back reaction from Q_A_^−^ to the donor side components of PSII, specifically the S_2_ state of the oxygen evolving complex (OEC) (Robinson and Crofts 1983; Vass et al. 1999). Most of the existing ChlF-based techniques apply STFs either from a measuring head in direct contact with the leaf surface, or from a few cm distance. *F*_m−ST_ is then recorded from a dark-adapted sample with an oxidized electron transport chain (Vernotte et al. 1979; Schansker et al. 2014). This allows standardized examination and modeling of ChlF relaxation kinetics in the dark (Vass et al. 1999). However, these requirements are impractical under conditions of ambient illumination, specifically when the presence of light is required for manifestation of stress conditions in the targeted plants. One such example is temperature stress, where low temperature enhances the photodamage effects of excess light (Pieruschka et al. 2010). In addition, the conventional fluorometric techniques may not provide sufficient resolution and throughput to capture highly dynamic regulation of photosynthesis.

The light-induced fluorescence transient (LIFT) method probes PSII from a distance using subsaturating (actinic) measuring flashlets in fast repetition rate (FRR) (Kolber et al. 1998; Osmond et al. 2017). In contrast to other techniques, no separate saturating flash is required in the LIFT method because the FRR probe flashlets are used directly for that purpose. The short measuring time of 0.2 s allows integration into automated systems for phenotyping in high spatio-temporal resolution. Following application in marine research (Kolber et al. 1998; Suggett et al. 2001; Oxborough et al. 2012; Robinson et al. 2014), a stationary LIFT system was installed for monitoring plant canopy from a distance of 50 m using laser excitation (Pieruschka et al. 2010, 2014; Raesch et al. 2014). Operating efficiency of PSII (*F*_q_′/*F*_m_′) measured with this previous LIFT system correlated well with pulse amplitude modulation (PAM) measurements (*R*^2^ = 0.89) and CO_2_ assimilation rates (*R*^2^ = 0.94) (Ananyev et al. 2005a; Pieruschka et al. 2010, 2014).

Here, we evaluated a newly developed LIFT device for its capacity to yield robust fluorometric parameters useful in plant phenotyping. Parameters as maximum ChlF induced by FRR flash (*F*_m−FRR_) and Q_A_^−^ reoxidation efficiency in 0.65 ms (*F*_r1_/*F*_v_) and 120 ms (*F*_r2_/*F*_v_) relaxation phases were introduced. The parameters were determined in isolated thylakoids and intact plants subjected to different treatments (electron transport inhibitors, anaerobiosis, or light) and approximated well-established ChlF parameters.

## Materials and methods

### Plant cultivation

In total, 36 spinach (*Spinacia oleracea*) plants of genotype *Matador* were grown in the greenhouse in Jülich, Germany, under 16-h/8-h day/night cycle at 20 °C/18 °C. Light intensity was kept automatically between 60 and 300 µmol photons m^−2^ s^−1^ using additional lamps or shading nets. 400-mL pots were filled with a turf-clay substrate (ED73, Einheitserdewerke, Sinntal-Altengronau, Germany). Plants were watered automatically twice a day during cultivation. Measurements were performed using plants 28 or 32 days after sowing.

### Isolation of thylakoids and PSII enriched membrane particles

For isolation of spinach thylakoids and PSII-enriched thylakoid membrane particles (BBY particles), fresh spinach leaves were bought from a local supermarket in Szeged, Hungary, and prepared as described in Berthold et al. (1981). For measurements with LIFT and FL3000, the final concentration of thylakoids was adjusted to equivalent chlorophyll *a* concentration of 10 µM (~ 10 µg mL^−1^).

### DCMU and DBMIB treatment

To manipulate the ChlF relaxation kinetics in thylakoid samples, we used 3-(3,4-dichlorophenyl)-1,1-dimethylurea (DCMU) and 2,5-dibromo-5-methyl-6-isopropyl-benzoquinone (DBMIB), which inhibit selectively the reoxidation of Q_A_^−^ in PSII and of PQH_2_ at the cytb_6_f complex, respectively (Lazár et al. 2001; Kurisu et al. 2003). A thylakoid suspension of 3 mL was transferred to transparent plastic cuvettes. After 5-min dark-adaption, DCMU and DBMIB were added to final concentration of 5 µM (1.17 µg mL^−1^) and 0.66 µM (0.213 µg mL^−1^), respectively. Samples were stirred manually and followed by either LIFT or FL3000 measurements using FRR flash for 0.75 ms (FRRF_0.75ms_) or STF, respectively. The number of technical replicates was 3–5. In addition, intact leaves were treated with DCMU to observe ChlF induction curves under conditions of blocked electron transport between Q_A_ and Q_B_. Plants were dark-adapted overnight, then fully expanded leaves were left untreated or were subjected to 200 µM DCMU in 50 mL Milli-Q water (Tóth et al. 2005). The control was left untreated because a control with 1% ethanol in distilled water showed no effect on *F*_m_ and little effect on the ChlF rise compared to untreated leaves (Tóth et al. 2005). However, no ethanol was used in the DCMU solution to avoid possible side effects (Haldimann and Tsimilli-Michael 2005). DCMU was grinded to powder in order to dissolve it better in water. In the dark, one leaf per plant was left for 6 h in DCMU solution, then wiped and left for 30 min in the air. Measurements on attached leaves were done using 5 FRRFs_0.75ms_ followed by one MTF for 750 ms (MTF_750ms_). Only the first of the 5 FRRFs_0.75ms_ is shown in the result section. Measurements were replicated with six different plants.

### Anaerobic treatment under nitrogen atmosphere

Oxygen depletion inhibits the plastid terminal oxidase (PTOX), which normally keeps PQ in an oxidized state in the dark (Bohme et al. 1971; Cournac et al. 2000; Feilke et al. 2014). Anoxic treatment was used to manipulate the level of PQ reduction non-invasively in living plants (Tóth et al. 2007b). The anoxic atmosphere was maintained in the LI-COR 6400 transparent 2 × 3 cm chamber head (LI-COR, Inc., Nebraska USA) using nitrogen gas (N_2_). Air inflow into the chamber came either from the ambient air (as control, with 400 ppm CO_2_) or from N_2_ gas supply without oxygen (containing < 1.5 ppm CO_2_). The air flow rate during the measurements was 300 µmol air s^−1^, and the block temperature of the LI-COR was kept at 20 °C. Prior to measurements, plants were dark-adapted overnight. A fully expanded leaf was inserted into the chamber and measured with the LIFT instrument through the transparent front window. Measurements were started after 5-min exposure to control or N_2_ atmosphere using 5 FRRFs_0.75ms_ followed by one MTF_750ms_. After another 5 min, measurements were repeated using 5 FRR flashes for 2.5 ms (FRRFs_2.5ms_). Each first flash of the 5 FRRFs_0.75ms_ and 5 FRRFs_2.5ms_ is shown the result section. Measurements were replicated with six different plants.

### Light response curves

To study electron transport kinetics of light-adapted plants, control plants of the N_2_ atmosphere experiment were subjected to increasing levels of blue light provided by the LED (445 nm) light source of the LIFT instrument. The size of the illumination spot was around 3 cm^2^. A light response measurement consisted of a total of 160 FRRF_0.75ms_ triggered at a 5-s interval. At every light intensity (30, 100, 300, 700 µmol photons m^−2^ s^−1^), ChlF was monitored over a period of 200 s by applying 40 consecutive FRRF_0.75ms_. Light response curves were replicated with six different plants.

### Fluorescence measurements

Different methods have been developed to separate absolute ChlF intensity and background radiation from relative changes of ChlF yield due to Q_A_ reduction. This allows comparison of minimal ChlF (*F*_o_ in dark-adapted state, and *F*′ in background light) and maximal ChlF (*F*_m_ in dark-adapted state, and *F*_m_′ in background light) at initial redox state of Q_A_ and when Q_A_ is fully reduced at the end of a saturating flash, respectively (Schreiber et al. 1986a; Strasser et al. 1995; Kolber et al. 1998). Two different fluorometers as described below were used in this study. The LIFT method requires no additional saturating light pulse besides the measuring flashlets. Therefore, it is referred to as a modulated method. In contrast, the FL3000 fluorometer uses weak measuring pulses and a strong excitation flash. Therefore, it is referred to as a double-modulated method.

#### FL3000 measurements

ChlF relaxation after a STF was monitored by weak, non-actinic measuring flashes in increasing time intervals (Trtilek et al. 1997; Vass et al. 1999). These double-modulated ChlF measurements were performed with a FL3000 fluorometer (Photon Systems Instruments Ltd., Brno, Czech Republic) (Trtilek et al. 1997). The instrument is equipped with red LEDs (639 nm) for both actinic (20 µs with an excitation power of 1020 µmol photons m^−2^ s^−1^) and weak, non-actinic measuring flashes of 8 µs, with a measuring delay of 7 µs. Changes in ChlF yield can be measured in a very broad time range, from 100 µs to 100 s. Within this time range, reoxidation of Q_A_^−^ by both forward and backward reactions can be studied (Vass et al. 1999). The reoxidation phase after the STF usually shows three relaxation phases with corresponding *τ*_1_, *τ*_2_, and *τ*_3_ time constants.

#### LIFT measurements

**Fig. 1:**
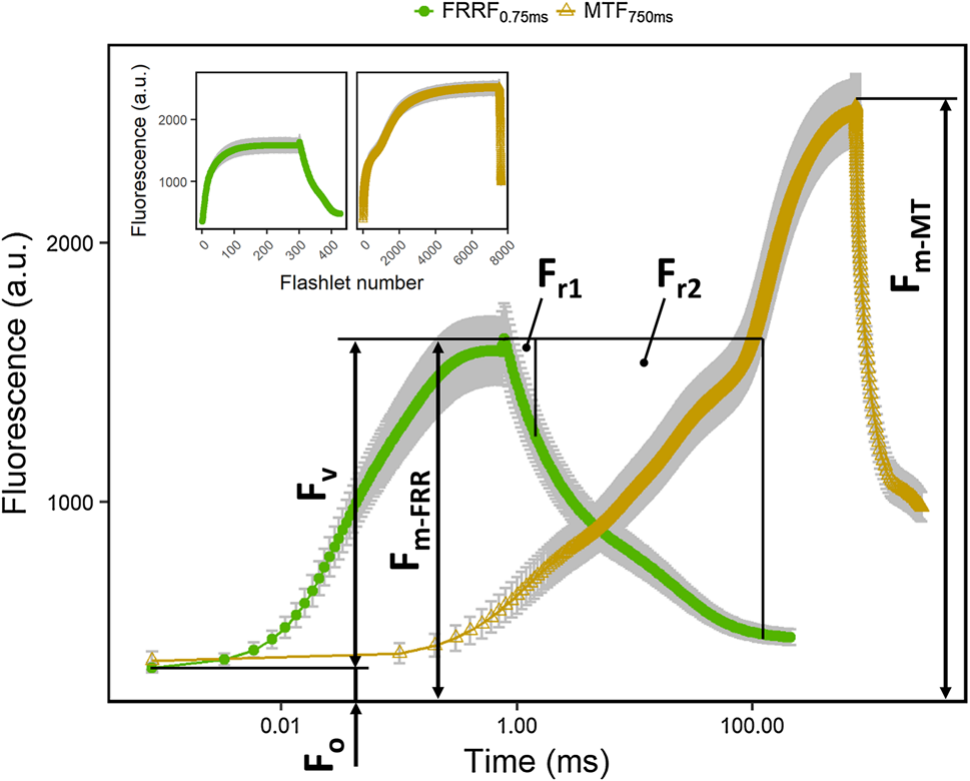
Chlorophyll fluorescence transients of spinach leaves induced by fast repetition rate flash (FRRF_0.75ms_) and multiple turnover flash (MTF_750ms_) are presented on a logarithmic time scale. The FRRF_0.75ms_ protocol (green circles) consists of 300 subsaturating flashlets (40,000 µmol photons m^−2^ s^−1^) in the first 0.75 ms to saturate maximum fluorescence (*F*_m−FRR_). Minimum fluorescence (*F*_o_), *F*_m−FRR_, and variable fluorescence (*F*_v_) were used for calculation of the quantum efficiency of photosystem II (*F*_v_/*F*_m_) in the dark-adapted state. The excitation flash is followed by 127 flashlets at exponential decreasing repetition rate resulting in chlorophyll fluorescence relaxation. The area above this relaxation curve was restricted by two time period (*r*_1_ = 0.65 ms and *r*_2_ = 120 ms after *F*_m−FRR_ was reached) resulting in two relaxation phases (*F*_r1_ and *F*_r2_). The areas were normalized with the corresponding time period and with *F*_v_ in order to retrieve the reoxidation efficiency of the primary quinone acceptor (Q_A_) in the given relaxation phases (*F*_r1_/*F*_v_ and *F*_r2_/*F*_v_). The MTF_750ms_ (yellow triangles) protocol consists of 7500 subsaturating flashlets (1000 µmol photons m^−2^ s^−1^) in the first 750 ms and induced the maximum fluorescence (*F*_m−MT_). Inset: Chlorophyll fluorescence transients against flashlet number of FRRF_0.75ms_ and MTF_750ms_ are shown. Error bars show standard deviation of the mean (*n* = 6 plants). (Color figure online)

The newly developed compact LIFT instrument (Version LIFT-REM, Soliense Inc., New York, USA) is equipped with a blue LED (445 nm) excitation source. Excitation protocols composed of up to 7500 flashlets are used to manipulate the level of photosynthetic activity and ChlF (Fig. 1). ChlF emission is detected at 685 (± 10) nm. The LIFT device monitors any background signal in the detector range during inter-flashlet periods and subtracts this signal from the in-flashlet ChlF signal. The ChlF yield is internally normalized against excitation power of each flashlet to correct for smaller fluctuations. Flashlet excitation power along the entire FRR excitation phase is kept at a constant level. This was verified by observing a flat fluorescence transient using a fluorescence standard with constant quantum yield of fluorescence.

All measurements were done from a 0.6 m distance with flashlet length of 1.6 µs. The three used FRR flashes differ in the length of flashlet interval and ChlF induction phase (Table 1). FRRF_0.75ms_ consists of 300 flashlets with a 2.5-µs interval summing up to the 0.75 ms induction phase. The ChlF relaxation phase after the FRRF_0.75ms_ consists of 127 flashlets. The interval between those flashlets increases exponentially with

$${j_i}={10^{1.28+0.0215 \times i}}\mu {\text{s,}}$$

where *j*_*i*_ is the interval length of the *i*th flashlet. For the DCMU and DBMIB experiments, the excitation power was approximately 20,000 µmol photons m^−2^ s^−1^ for the ChlF induction phase using FRRF_0.75ms_. Due to the restricted excitation power of the LEDs, the FRRF_0.75ms_ lasts longer than a proper STF in order to get *F*_m−FRR_ saturated. For intact plants, the excitation power was 40,000, 24,000, and 1000 µmol photons m^−2^ s^−1^ for the ChlF induction phase using FRRF_0.75ms_, FRRF_2.5ms_, and MTF_750ms_, respectively. The interval between the flashlets for MTF_750ms_ was extended from 2.5 to 100 µs due to exhausting of LED at longer flashes. This resulted in the lower excitation power. Excitation power was measured at 1% duty cycle using a 5-s calibration flash measured by a quantum sensor (LI-190R, LI-COR, Inc.) and then extrapolated to 100%. For application of constant actinic light, the intensity of the blue LIFT LED in DC mode was calibrated using a quantum sensor (LI-190R, LI-COR, Inc.) at 0.6-m distance.

**Table 1:** Different excitation protocols are shown: Fast repetition rate flash for 0.75 ms (FRRF_0.75ms_) and for 2.5 ms (FRRF_2.5ms_) as well as saturating multiple turnover flash for 750 ms (MTF_750ms_). In the induction phase, flashlet interval is constant for given amount of flashlets and interval, while it increases exponentially in the relaxation phase to allow reoxidation of the primary quinone acceptor (Q_A_). Flashlet length is always 1.6 µs and has in all flashes the same specified excitation power

Excitation protocol	Induction phase (ms)	Number of flashlets in induction phase	Flashlet interval in induction phase (µs)	Relaxation phase (ms)	Number of flashlets in relaxation phase	Flashlet length (µs)
FRRF_0.75ms_	0.75	300	2.5	209	127	1.6
FRRF_2.5ms_	2.5	1000	2.5	209	127	1.6
MTF_750ms_	750	7500	100	1975	127	1.6

#### Analysis of LIFT and FL3000 raw data

For the calculation of *F*_v_/*F*_m_, the variable ChlF (*F*_V_) is the difference between *F*_m_ and *F*_o_. In the LIFT analysis, *F*_m_ is represented by *F*_m−FRR_ as the averaged ChlF yield of 301st–302nd flashlet. *F*_o_ is the ChlF yield of the first flashlet (Fig. 1). In the FL3000 analysis, *F*_m_ is represented by *F*_m−ST_ and measured 0.15 ms after the STF. *F*_o_ is measured before the STF. The Q_A_^−^ reoxidation efficiency is calculated from the ChlF relaxation kinetics as follows:

$${{{F_{r1,2}}} \mathord{\left/ {\vphantom {{{F_{r1,2}}} {{F_V}}}} \right. \kern-0pt} {{F_V}}}={{\left( {{F_V} \times {t_{1,2}} - \sum {{F_i} \times {j_i}} } \right)} \mathord{\left/ {\vphantom {{\left( {{F_V} \times {t_{1,2}} - \sum {{F_i} \times {j_i}} } \right)} {\left( {{F_V} \times {t_{1,2}}} \right)}}} \right. \kern-0pt} {\left( {{F_V} \times {t_{1,2}}} \right)}}$$

Here, *F*_*i*_ is the ChlF yield in the relaxation phase at flashlet *i. F*_*i*_ is multiplied by *j*_*i*_ and summed up to represent the area of ChlF relaxation up to *t*_1_ = 0.65 ms (for F_r1_) and *t*_2_ = 120 ms (for *F*_r2_). In case of the FL3000 data, the time points for *t*_1_ and *t*_2_ were 0.52 ms and 100 ms after the STF, respectively, due to different relaxation protocols. The light-adapted states of *F*_o_, *F*_m−FRR_, *F*_v_, and *F*_r1,2_ are denoted as *F*′, *F*_m−FRR_′, *F*_q_′, and *F*_r1,2_′, respectively.

### Statistics

Analysis of variance (ANOVA) was used to calculate significant differences (*p* < 0.05) followed by Tukey’s test for pairwise comparison. Due to the small sample size (*n* < 7), normal distribution and homogeneity of variance were assumed. Analysis was done by R program using the *multcomp* package.

## Results

Photosynthetic characteristics were studied by measuring light-induced ChlF transients using both the modulated LIFT and the double-modulated FL3000 device with an emphasis on the properties of electron transport from PSII towards PSI.

### Determination of *F*_m_ levels and electron transport kinetics

**Fig. 2:**
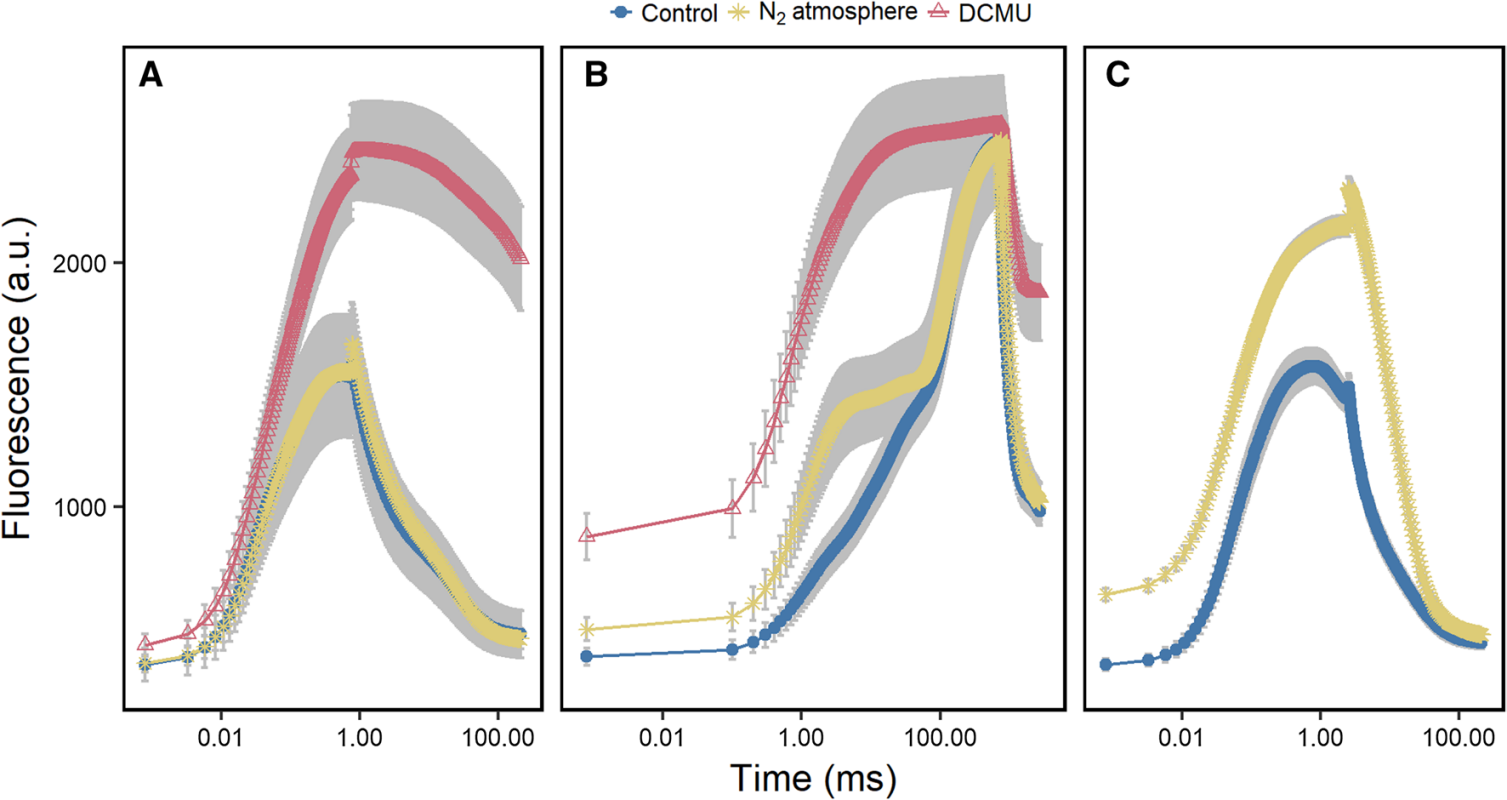
Dark-adapted spinach leaves were subjected to a 3-(3,4-dichlorophenyl)-1,1-dimethylurea (DCMU) treatment and nitrogen (N_2_) atmosphere, which prevent reoxidation of the primary quinone acceptor (Q_A_), and plastoquinone (PQ) pool in the dark, respectively. Under those treatments, fast repetition rate flash for 0.75 ms (FRRF_0.75ms_, **a**), multiple turnover flash (MTF_750ms_, **b**), and fast repetition rate flash for 2.5 ms (FRRF_2.5ms_, **c**) were used to study chlorophyll fluorescence induction and relaxation. FRRF_0.75ms_ was performed after 5 min in control or N_2_ atmosphere (for DCMU treatment see “Materials and methods” section). MTF_750ms_ was performed after FRRF_0.75ms_. FRRF_2.5ms_ was performed after additional 5 min in control or N_2_ atmosphere. Chlorophyll fluorescence transients are presented on a logarithmic time scale. Error bars showing standard deviation of the mean (*n* = 6 plants)

We used the FRRF_0.75ms_ and MTF_750ms_ protocol to study the Induction of *F*_m−FRR_ and *F*_m−MT_, respectively (Table 1). The *F*_m−FRR_ in control leaves was significantly lower than *F*_m−MT_ (Figs. 1, 2a, b). Under control conditions, *F*_m−FRR_ saturated earliest at about 0.25 ms depending on the excitation power (Fig. S1). At the end of the induction phase, a small peak of ChlF occurs pointing towards a minor ChlF quenching during the high excitation power of the FRRF_0.75ms_. These spikes contributed to *F*_m−FRR_ resulting in *F*_v_/*F*_m_ values independent of the excitation power. In contrast, *F*_m−MT_ in intact leaves was reached after 750 ms and showed the same *F*_m−MT_ as in the presence of DCMU (Fig. 2b).

**Fig. 3:**
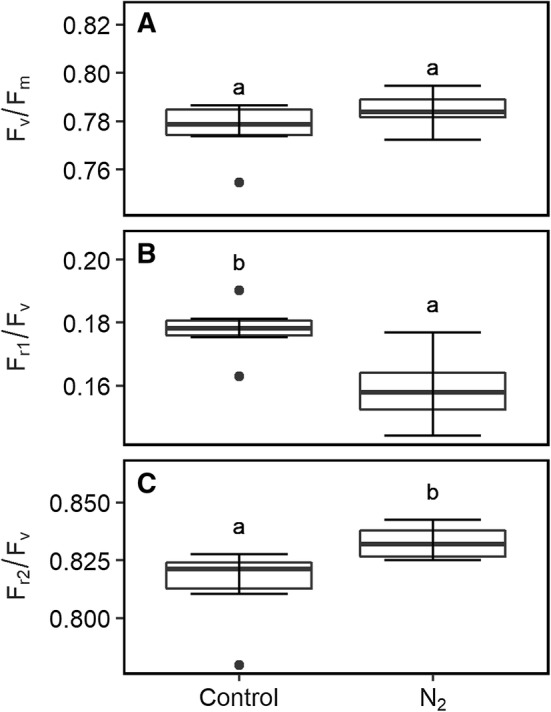
Boxplot of quantum efficiency of the photosystem II (*F*_v_/*F*_m_, **a**), efficiency of primary quinone acceptor (Q_A_) reoxidation in 0.65 ms (*F*_r1_/*F*_v_, **b**), and 120 ms (*F*_r2_/*F*_v_, **c**) relaxation phases of dark-adapted attached spinach leaves are shown. Measurements took place 5 min after exposure to control or nitrogen (N_2_) atmosphere. Parameters were obtained using fast repetition rate flash (FRRF_0.75ms_) of the light-induced fluorescence transient (LIFT) instrument. Box represents inter-quartile range, bold horizontal bar the median, the discontinuous lines the upper and lower quartile, and outlier data points (> 1.5 × inter-quartile range) are depicted by a point (*n* = 6 plants). Boxes labeled with different letters differ significantly from each other according to Tukey’s multiple comparisons of means

We studied ChlF relaxation on attached leaves in the dark by using FRRF_0.75ms_ under control and N_2_ atmosphere. The absence of O_2_ in the latter condition prevents reoxidation of the PQ pool in the dark (Tóth et al. 2007b). After 5 min in the N_2_ atmosphere, ChlF relaxation phase was significantly altered compared to control conditions (Fig. 2a). While *F*_v_/*F*_m_ did not change under control and N_2_ atmosphere, *F*_r1_/*F*_v_ in the N_2_ atmosphere decreased significantly to 0.16 (± 0.01) compared to 0.18 (± 0.01) in the control (Fig. 3). In contrast, *F*_r2_/*F*_v_ was significantly increased in the N_2_ atmosphere compared to the control. Notably, also the ChlF induction phase of MTF_750ms_ differed in N_2_ atmosphere compared to control (Fig. 2b). Following MTF_750ms_, we kept the plants for additional 5 min under the control or N_2_ atmosphere. This allowed S-states of the OEC to relax in the dark (Kolber et al. 1998), whereas the PQ pool remained reduced in the N_2_ atmosphere. Then a subsequent measurement using FRRF_2.5ms_ was initiated. *F*_m−FRR_ in the control treatment was reached at around 750 µs and then the ChlF signal started to decline (Fig. 2c). In the N_2_ atmosphere, the ChlF level continued to increase during the FRRF_2.5ms_ without reaching saturation. This resulted in a significantly increased *F*_m−FRR_ relative to the control. In addition, *F*_o_ levels were higher in the N_2_ atmosphere compared to control in the subsequent flashes (Fig. 2b, c). This led to significantly lowered *F*_v_/*F*_m_ under N_2_ atmosphere using FRRF_2.5ms_ (*p* value < 0.001). In summary, *F*_m−MT_ induction in untreated leaves using MTF_750ms_ was confirmed by the same *F*_m_ induced in DCMU-treated leaves. Full saturation of *F*_m−FRR_ level was confirmed using FRRF_2.5ms_ under two conditions: (1) plants were in controlled, aerobic conditions (PQ pool was oxidized); and (2) the leaf was fully dark-adapted (OEC mainly in the S_1_-state) (Delosme and Joliot 2002). The influence of increased PQ pool reduction was reflected in decreased *F*_r1_/*F*_v_, and increased *F*_o_ and *F*_m−FRR_.

### Comparison of electron transport kinetics measured by the LIFT and FL3000 device

**Fig. 4:**
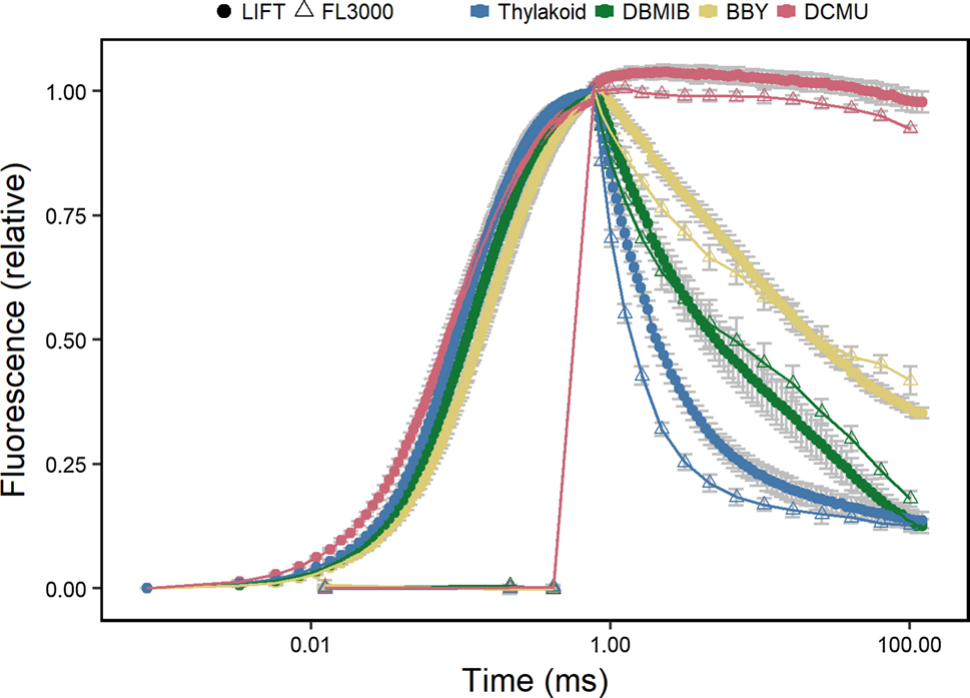
Chlorophyll fluorescence transients of isolated spinach thylakoids and photosystem II particles (BBY) are presented on a logarithmic time scale. The measurements were performed either by the light-induced fluorescence transient (LIFT) device (closed circles) or with the double-modulated FL3000 fluorometer (open triangles). Thylakoid samples (10 µg chlorophyll/mL) were either untreated, or treated with 5 µΜ 3−(3,4-dichlorophenyl)-1,1-dimethylurea (DCMU) or 0.66 µM 2,5-dibromo-5-methyl-6-isopropyl-benzoquinone (DBMIB). Chlorophyll fluorescence signals are double normalized so that the signal starts from 0 for measured minimum fluorescence (*F*_o_), and has a total amplitude of 1. Chemicals were added in the dark and samples were dark-adapted for 3 min before measurement. Error bars show standard deviation (*n* = 5, except DCMU FL3000 and DBMIB FL3000 *n* = 3)

**Fig. 5:**
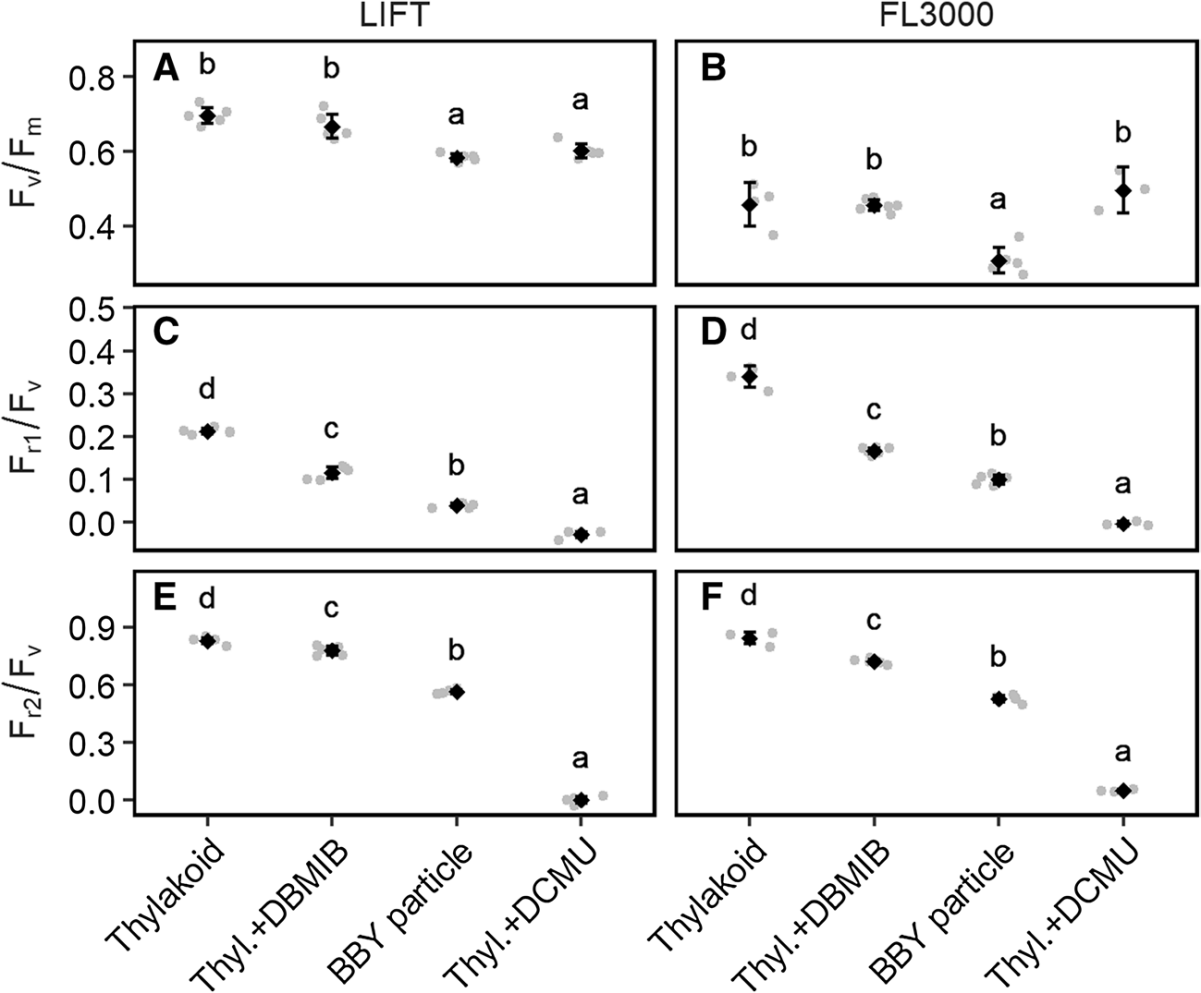
Comparison of photosystem II quantum efficiency (*F*_v_/*F*_m_, **a, b**) and efficiency of primary quinone acceptor (Q_A_) reoxidation in 0.65 ms (*F*_r1_/*F*_v_, **c, d**), and 120 ms (*F*_r2_/*F*_v_, **e, f**) relaxation phases in dark-adapted state acquired by light-induced fluorescence transient (LIFT) and double-modulated FL3000 fluorometer. Measurements were carried out on isolated spinach thylakoids and BBY particles. Thylakoid samples (10 µg chlorophyll/mL) were either untreated or treated with 5 µΜ 3−(3,4-dichlorophenyl)-1,1-dimethylurea (DCMU) or 0.66 µM 2,5-dibromo-5-methyl-6-isopropyl-benzoquinone (DBMIB), resulting in different chlorophyll fluorescence relaxations as shown in Fig. 4. Black diamonds show mean values and error bars indicate the 95% confidence intervals. Individual data points are depicted by a grey point (*n* = 5, except DCMU FL3000 and DBMIB FL3000 *n* = 3). Means labeled with different letters differ significantly from each other according to Tukey’s multiple comparisons of means

We compared the ChlF relaxation kinetics acquired by the modulated LIFT to those of the double-modulated FL3000 device. For that purpose, we treated the thylakoids with different electron transport inhibitors. The two methods resulted in similar ChlF relaxation curves (Fig. 4). The *F*_v_/*F*_m_ values calculated from the LIFT measurements ranged between 0.58 (± 0.01) for BBY and 0.7 (± 0.02) for thylakoids (Fig. 5a). The FL3000 device showed generally lower *F*_v_/*F*_m_ values: 0.31 (± 0.04) for BBY particles and 0.49 (± 0.02) for thylakoids (Fig. 5b). In BBY particles, electron transport is impaired after the Q_B_ site since the PQ pool is partly, and the PSI fully removed (Berthold et al. 1981). DBMIB binds to the cytb_6_f complex, which blocks the reoxidation of the PQ pool (Bohme et al. 1971). Consequently, BBYs and DBMIB-treated thylakoids showed slower ChlF relaxation kinetics compared to thylakoids, resulting in significantly lower *F*_r1_/*F*_v_ and *F*_r2_/*F*_v_ values in both methods (Fig. 5c–f). *F*_r1_/*F*_v_ for the LIFT device ranged from 0.21 (± 0.008) for thylakoids to 0.04 (± 0.008) for BBY (Fig. 5c). The *F*_r1_/*F*_v_ values calculated from the FL3000 measurements were in general higher (e.g., 0.34 (± 0.025) for thylakoids and 0.1 (± 0.013) for BBY, Fig. 5d) but showed the same tendency as in the LIFT measurements. *F*_r2_/*F*_v_ showed increasing difference between the control and treated samples reflecting impaired electron transport (Fig. 5e, f). In summary, *F*_r1_/*F*_v_ and *F*_r2_/*F*_v_ measured with both devices responded specifically to the treatments which block electron transport at different steps.

### Electron transport kinetics measured under ambient light

**Fig. 6:**
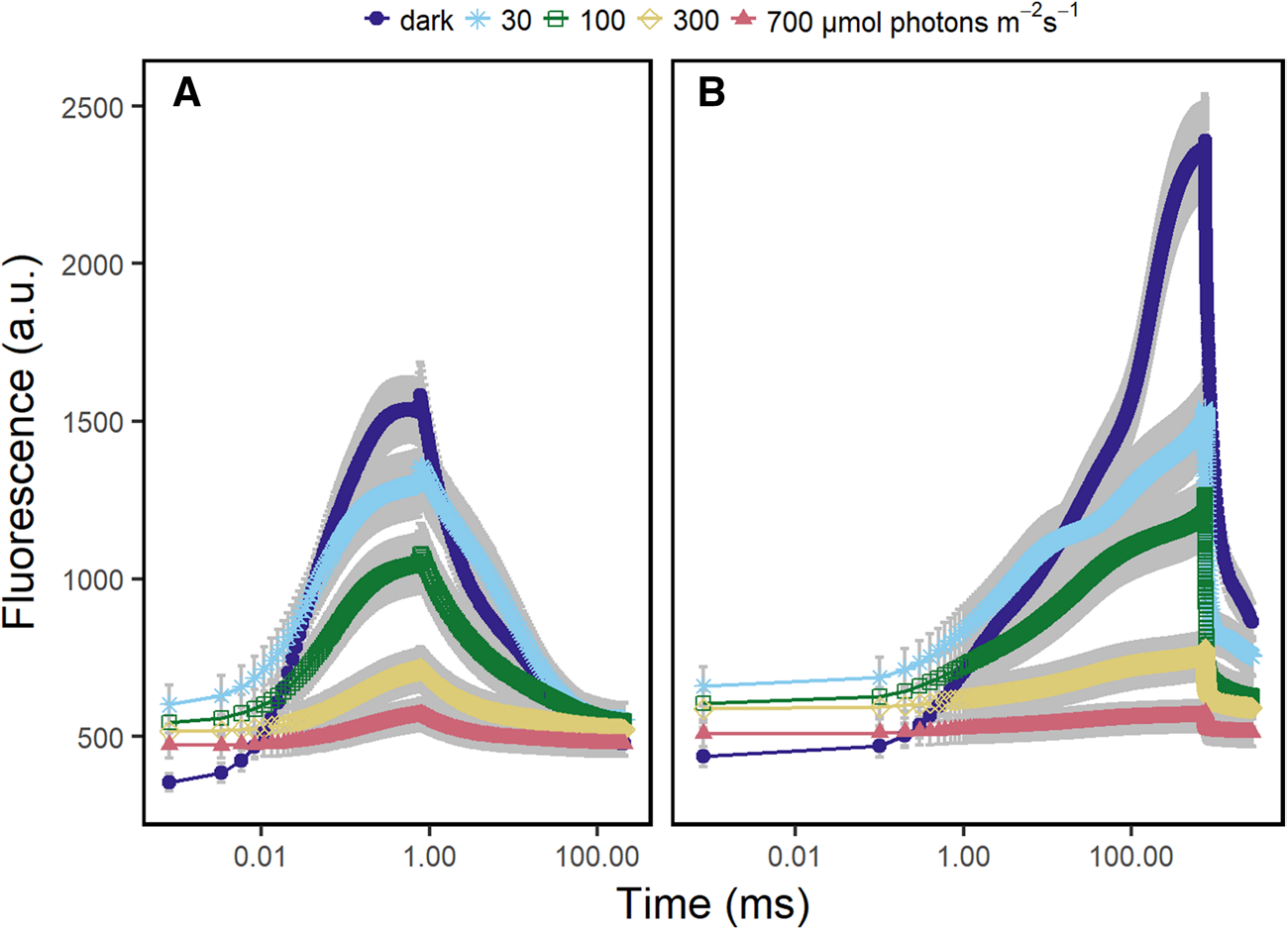
Chlorophyll fluorescence response of attached spinach leaves measured under different intensities of background irradiance by the light-induced fluorescence transient (LIFT) instrument. Leaves were exposed to 0 (dark-adapted), 30, 100, 300, and 700 µmol photons m^−2^ s^−1^ blue light (445 nm). Fast repetition rate flash (FRRF_0.75ms_, **a**) and multiple turnover flash (MTF_750ms_; **b**) were performed on dark-adapted samples and after reaching the steady state at each light intensity (after 3 min). Chlorophyll fluorescence transients are presented on a logarithmic time scale. Error bars show the standard error of the mean (*n* = 6 plants)

We measured light-response curves on attached spinach leaves in order to follow the light saturation of electron transport rate. F′ initially increased with increasing light intensities, but this increase was then reversed, most likely due to NPQ formation (Fig. 6a). Simultaneously, *F*_m−FRR_′ decreased in response to increasing light intensities due to NPQ formation. *F*_m−FRR_′ showed smaller absolute differences than *F*_m−MT_′ compared to the corresponding dark-adapted states (Fig. 6b).

**Fig. 7:**
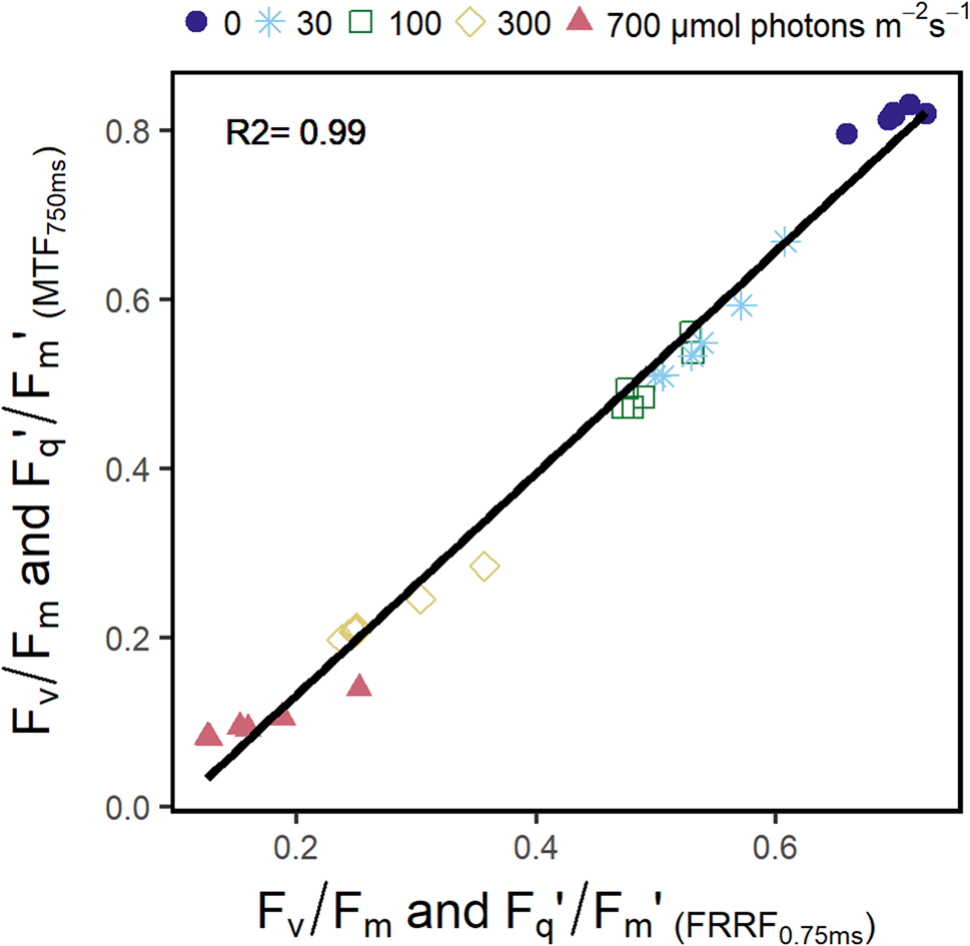
Correlation of quantum efficiency of the photosystem II in the dark-adapted state (*F*_v_/*F*_m_) and the light-adapted state (*F*_q_′/*F*_m_′) obtained by fast repetition rate flash (FRRF_0.75ms_) and multiple turnover flash (MTF_750ms_) during a blue-light response curve of spinach leaves. Maximum fluorescence (*F*_m_ in the dark and *F*_m_′ in the light) represents chlorophyll fluorescence yield of the averaged 300th and 301st flashlet of the FRRF_0.75ms_ respective the yield of the 7500th flashlet in case of the MTF_750ms_. Variable fluorescence (*F*_v_ in the dark or *F*_q_′ in the light) is the difference between *F*_m_ or *F*_m_′ and the chlorophyll fluorescence yield of the first flashlet (*F*_o_ in the dark or *F*′ in the light). The regression formula was *y* = − 0.13 + 1.31 × *x*. Measurements were performed using the light-induced fluorescence transient (LIFT) device (*n* = 6 plants)

**Fig. 8:**
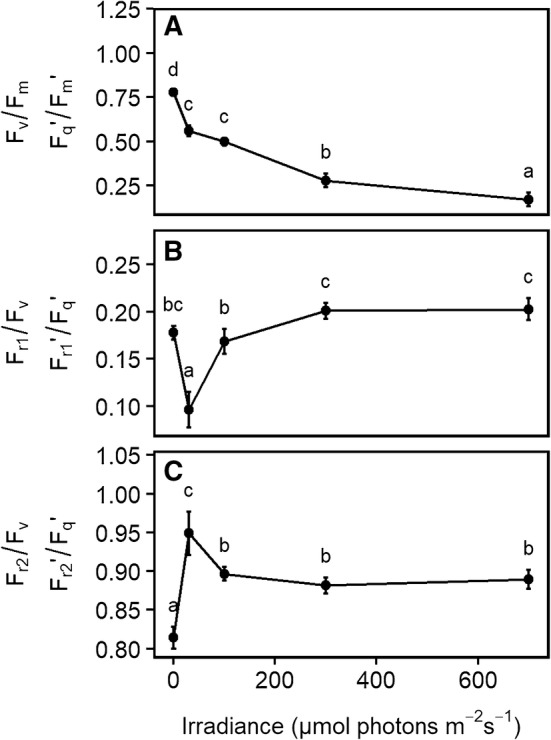
Quantum efficiency of the photosystem II (*F*_v_/*F*_m_ in the dark, and *F*_q_′/*F*_m_′ in the light, **a**) and efficiency of primary quinone acceptor (Q_A_) reoxidation in 0.65 ms (*F*_r1_/*F*_v_ in the dark, and *F*_r1_′/*F*_q_′ in the light, **b**,) and 120 ms (*F*_r2_/*F*_v_ in the dark, and *F*_r2_′/*F*_q_′ in the light, **c**) relaxation phases of attached spinach leaves were measured under different intensities of background irradiance. Parameters were acquired using fast repetition rate flash (FRRF_0.75ms_) of the light-induced fluorescence transient (LIFT) instrument. Black dots show mean values and error bars indicate the 95% confident interval (*n* = 6 plants). Means labeled with different letters differ significantly from each other according to Tukey’s multiple comparisons of means

The *F*_q_′/*F*_m_′ values we obtained with the FRRF_0.75ms_ protocol correlated highly with *F*_q_′/*F*_m_′ values we retrieved with the MTF_750ms_ protocol (*r*^2^ = 0.99) during the measurements of blue-light response curves (Fig. 7). This demonstrates that FRRF_0.75ms_ and MTF_750ms_ measurements result in basically the same parameters with the exception of an offset, at least under these standard conditions. Increasing light intensities resulted in a significant decrease in the *F*_q_′/*F*_m_′ (Fig. 8a). In contrast, *F*_r1_′/*F*_q_′ and *F*_r2_′/*F*_q_′ were less affected by the higher light intensities (Fig. 8b, c). Upon dark to light transition at 30 µmol photons m^−2^ s^−1^, *F*_r1_′/*F*_q_′ decreased from 0.18 (± 0.009) to 0.10 (± 0.023) and increased to 0.2 (± 0.01) at the last two light intensities. *F*_r2_′/*F*_q_′ increased from 0.81 (± 0.018) to 0.95 (± 0.035) and then was stabilized at 0.89 (± 0.02) at the higher light intensities. *F*_r1_′/*F*_q_′ measured in the light was not significantly different from dark-adapted values, with the exception of the initial light step at 30 µmol photons m^−2^ s^−1^. In summary, ChlF relaxation kinetics in the light were little affected by increasing light intensities and NPQ, whereas *F*_q_′/*F*_m_′ decreased.

## Discussion

We induced ChlF transients by using different LIFT-FRR excitation protocols (FRRF_0.75ms_ and MTF_750ms_) at 0.6 m distance (Fig. 1). *F*_v_/*F*_m_ and *F*_q_′/*F*_m_′ were highly correlated between the two protocols (Fig. 7). Furthermore, we characterized a range of photo-physiological properties with an emphasis on the kinetics of electron transport from PSII towards PSI. These kinetics are determined by the well-established architecture of photosynthetic linear electron transport chain and can be observed via ChlF relaxation reflecting the kinetics of Q_A_^−^ reoxidation (Vass et al. 1999). Efficiency of Q_A_^−^ reoxidation was assessed in 0.65 ms and 120 ms relaxation phases after the FRR excitation, expressed in the *F*_r1_/*F*_v_ and *F*_r2_/*F*_v_ parameter, respectively. These simple parameters reflect the overall reoxidation of Q_A_^−^ during the indicated time periods. In the light, *F*_r2_′/*F* reflected electron transport capacity from Q_A_ towards PSI and was far less sensitive to increasing light intensities than *F*′/*F*_m_′ (Fig. 8). The results provide additional information about electron transport, which are not reflected by the *F*_v_/*F*_m_ parameter.

### Maximum fluorescence

We demonstrated ChlF induction at 0.6 m distance by using the LIFT instrument on attached spinach leaves. *F*_m−MT_ in the control leaves was reached at 750 ms after multiple turnover of PSII reaction centers (Fig. 2b). That *F*_m−MT_ was indeed saturated by using MTF_750ms_, was confirmed by using the DCMU treatment, which showed the same ChlF level. DCMU inhibits Q_A_^−^ reoxidation and induces *F*_m−MT_ in intact leaves (Tóth et al. 2005). In contrast to *F*_m−MT_, *F*_m−ST_ is reached within 40 to 60 µs within one full turnover of the PSII reaction centers (Kolber et al. 1998; Nedbal et al. 1999; Belyaeva et al. 2014). The level of *F*_m−ST_ is about 50% lower compared to *F*_m−MT_ and is based on fully reduced Q_A_ (e.g., Samson and Bruce 1996; Schansker et al. 2014). The difference between *F*_−MT_ and *F*_m−ST_ is suggested to be caused by additional ChlF quenchers that are removed during multiple turnovers (Delosme 1967; Kalaji et al. 2017). In this study, the *F*_m−FRR_ induced by FRRF_0.75ms_ saturated at about 0.25 ms at highest excitation power of 40,000 µmol photons m^−2^ s^−1^ (Fig. S1). Within this time range, the OEC is already in the second turnover and Q_A_^−^ is once reoxidized by Q_B_ (Ananyev and Dismukes 2005b; Pérez-Navarro et al. 2016). The reoxidation of Q_A_^−^ by Q_B_ and Q_B_^−^ has time constants of 0.2 ms and 0.7 ms, respectively (Bowes and Crofts 1980; de Wijn and van Gorkom 2001; Tomek et al. 2001). The second time constants may vary with respect to the kinetics of H^+^ uptake by Q_B_^−^ (Petrouleas and Crofts 2005). The saturating behavior of *F*_m−FRR_ strongly indicates that photochemical processes stabilized within the FRRF_0.75ms_ (Fig. S1). In agreement, the derived *F*_v_/*F*_m_ values were independent from the used excitation power. We conclude that *F*_m−FRR_ reflected fully reduced Q_A_ mainly associated with Q_B_^−^.

Using the FRRF_2.5ms_ protocol, *F*_m−FRR_ declined after reaching a plateau under control conditions (Fig. 2c). This behavior was only observed when the PQ pool was oxidized and the sample was dark-adapted. Similarly, the polyphasic ChlF rise during a MTF of 15,000 µmol photons m^−2^ s^−1^ records a local ChlF maximum at about 2 ms (J-step) (Schreiber 1986a; Tóth et al. 2007a; Schansker et al. 2011). This ChlF peak appears only when the sample is in the S_1_-state, i.e., dark-adapted (Strasser 1997). In the ChlF decline, Q_A_ reduction is overcome by Q_A_^−^ reoxidation via the oxidized PSII primary donor (P680^+^), which accumulates during the slow S_3_–S_4_ transition of the OEC (Schansker et al. 2011; Kalaji et al. 2017). The phase of ChlF decline matches time wise with the formation of Q_B_^2−^ (Bowes and Crofts 1980; de Wijn and van Gorkom 2001). After 2 ms, electron delivery of the OEC proceeds and re-reduction of Q_A_, further accumulation of Q_B_^2−^ and exchange of Q_B_^2−^ by an oxidized PQ takes place (Petrouleas and Crofts 2005). These processes lead to an increasing ChlF signal during a MTF, known as thermal phase (Delosme 1967; Lazár 2006). It was shown before that PQ pool reduction leads to a higher ChlF signal by releasing non-photochemically quenched ChlF (Vernotte et al. 1979; Haldimann and Tsimilli-Michael 2005). In agreement, *F*_m−FRR_ increased without reaching saturation when the PQ pool was already reduced in the dark (Fig. 2c). Similarly, *F*_o_ under N_2_ atmosphere was also higher than in the control but was the same in the subsequent FRRF_2.5ms_ (*p* value = 0.403, data not shown) due to reoxidation of PQ pool during the flash. *F*_o_ yield was shown to be dependent on PQ redox state and can be recovered by far-red light pulse (Diner 1977; Hohmann-Marriott et al. 2010; Kalaji et al. 2017). However, this additional ChlF quenching is probably not directly controlled by the PQ pool (Tóth et al. 2005). It may be induced by the occupancy of the Q_B_-pocket or by conformational changes in the PSII complex (Falkowski et al. 2004; Schansker et al. 2014; Magyar et al. 2018; Prášil et al. 2018). This might explain that *F*_m−ST_ (when only Q_A_ is reduced) cannot surpass the ChlF level at the J-step (Schreiber 1986a). Another reason for the lower *F*_m−ST_ compared to the J-step might be that STF induced a quenching mechanism during the reduction phase of Q_A_. At the end of the FRRF_0.75ms_ induction phase, when changing from fast to low repetition rate flashlets, we noticed an instantaneous ChlF spike (Fig. S1). This indicates a fast-relaxing quenching mechanism. It was suggested earlier that carotenoid triplets quench ChlF within µs when operating with flashes at high excitation power (Schödel et al. 1999; Steffen et al. 2001; Braslavsky and Holzwarth 2012; Belyaeva et al. 2014). In summary, saturated *F*_m−FRR_ differs from *F*_m−ST_ in the reduction of Q_B_ to Q_B_^−^ while Q_A_ is fully re-reduced. *F*_m−FRR_ and *F*_m−ST_ are expected to be comparable since Q_B_^−^ is not known to quench any ChlF (Schansker et al. 2011). The saturation of *F*_m−FRR_ after 0.25 ms indicates that Q_B_^2−^ was not formed. The *F*_m−FRR_ differs from the J-step in the redox state of the OEC and the accumulation of Q_B_^2−^ which influence ChlF (see also Osmond et al. 2017). When reaching *F*_m−MT_, at least one additional quencher is removed increasing ChlF signal by a still unclear mechanism (Magyar et al. 2018; Prášil et al. 2018).

### Validation of electron transport kinetics

Anaerobiosis inhibits PQ pool reoxidation in the dark (Bohme et al. 1971; Cournac et al. 2000; Feilke et al. 2014). As expected, reduced PQ pool under N_2_ atmosphere affected ChlF relaxation and decreased *F*_r1_/*F*_v_ compared to control (Fig. 3b). ChlF relaxation was compared between the modulated LIFT-FRR and the double-modulated FL3000 device. The two devices measured similar qualitative responses comparable to earlier studies (Deák et al. 2014). However, Q_A_^−^ reoxidation was faster in the first milliseconds when measuring with the double-modulated FL3000 than the LIFT device (Fig. 4). This might be due to the shorter duration of the STF than FRRF_0.75ms_, where the latter reduced Q_B_ already. In addition, the FRR flashlets have an actinic effect, which partially reduce Q_A_ and slow down the Q_A_^−^ reoxidation kinetics. The STF of the FL3000 device with 1000 µmol photons m^−2^ s^−1^ was probably not saturating resulting in the lower *F*_v_/*F*_m_ values compared to the values derived by the LIFT device. In summary, a wide range of impaired electron transport processes were detected using the ChlF relaxation parameters *F*_r1_/*F*_v_ and *F*_r2_/*F*_v_ derived either by the LIFT or the FL3000 device.

### Measurements in the light and effect on electron transport kinetics

In the dark, the *F*_m−FRR_ yield was linked to PQ redox state and S-state of the OEC. The situation is different in the light because NPQ occurs and S-states are randomized. *F*_m−FRR_′ was interpreted as equilibrium of Q_A_ reduction, NPQ and subsequent Q_A_^−^ reoxidation (Fig. 6a, Osmond et al. 2017). Accordingly, *F*_m−MT_′ in the light does not reach saturation because NPQ limits light harvesting and electron transport quenches ChlF very efficiently (Loriaux et al. 2013). The MTF_750ms_ with rather low excitation power (1000 µmol photons m^−2^ s^−1^) limited *F*_m−MT_′ saturation additionally. However, *F*_q_′/*F*_m_′ derived by FRRF_0.75ms_ during the measurement of blue-light response curve correlated highly with *F*_q_′/*F*_m_′ derived by MTF_750ms_ (*R*^2^ = 0.99) (Fig. 7). Correspondingly, *F*_q_′/*F*_m_′ derived with a previous LIFT device using FRR flashes was shown to be well-correlated to the values measured by a PAM device (*R*^2^ = 0.89) (Pieruschka et al. 2010, 2014). Comparable results were also shown by Samson et al. (1999) who carried out a similar experiment using STF and MTF. Dark-light transition at 30 µmol photons m^−2^ s^−1^ was clearly separated based on the transient shape and the derived *F*_r1_′/*F*_q_′ and *F*_r2_′/*F*_q_′ values (Fig. 8). The strong initial response upon illumination appears to represent transition from the dark-adapted state of inactive electron transport to a light-stimulated state of engaged electron transport, e.g., activation of RUBISCO in the first few minutes of light acclimation (Kono and Terashima 2014). In conclusion, *F*_q_′/*F*_m_′ derived by FRRF_0.75ms_ approximate *F*_q_′/*F*_m_′ values derived by STF or MTF.

The *F*_m−ST_ and *F*_m−MT_ signal, along with *F*_v_/*F*_m_ and NPQ, are the most common photosynthetic parameters used in ChlF-assisted plant phenomics (Furbank and Tester 2011). These properties are relatively easy to measure by using existing ChlF techniques, and they are extremely sensitive to a range of photo-physiological properties of plants. At the same time, the *F*_m_ responses are rather non-specific, requiring additional information to identify the affected photosynthetic mechanisms (Kalaji et al. 2014). In addition, their direct responses to irradiance levels require that these parameters are measured under well-defined light conditions (generally in the dark, with pre-defined periods of dark adaptation), limiting their applications as reporters of physiological conditions under highly variable, natural light conditions. However, the properties of the photosynthetic electron transport from Q_A_ towards PSI (expressed as *F*_r2_′/*F*_q_′) remain well-constrained under different ambient light intensities (Fig. 8c). The possibility for automation and measurements in the light using the LIFT method will make it possible to monitor the dynamics of photosynthetic traits under natural conditions.

## Conclusion

Simultaneous measurements of *F*_v_/*F*_m_ (respective *F*_q_′/*F*_m_′) and the kinetics of electron transport between PSII towards PSI expressed as *F*_r1,2_/*F*_v_ (respective *F*_r1,2_′/*F*_q_′) parameter provided more detailed information about the photosynthetic apparatus detecting differences in a wide range of physiological conditions. Performing these measurements non-invasively with high time resolution under natural environmental conditions has the potential to improve the efficacy of the photosynthetic phenotyping, while contributing to the advancement of knowledge about photosynthesis and its regulation.

## Electronic supplementary material

Below is the link to the electronic supplementary material.

Supplementary material 1 (DOCX 232 KB)
