# Supplementary material for: Maximum fluorescence and electron transport kinetics determined by light-induced fluorescence transients (LIFT) for photosynthesis phenotyping
Source: Photosynth Res. 2018 Oct 24;140(2):221–33. doi: 10.1007/s11120-018-0594-9 (PMC6548062; doi:10.1007/s11120-018-0594-9)
Supplement: Supplementary file 1 — Supplementary material 1 (DOCX 232 KB) [file 11120_2018_594_MOESM1_ESM.docx]

## Supplemental Figures


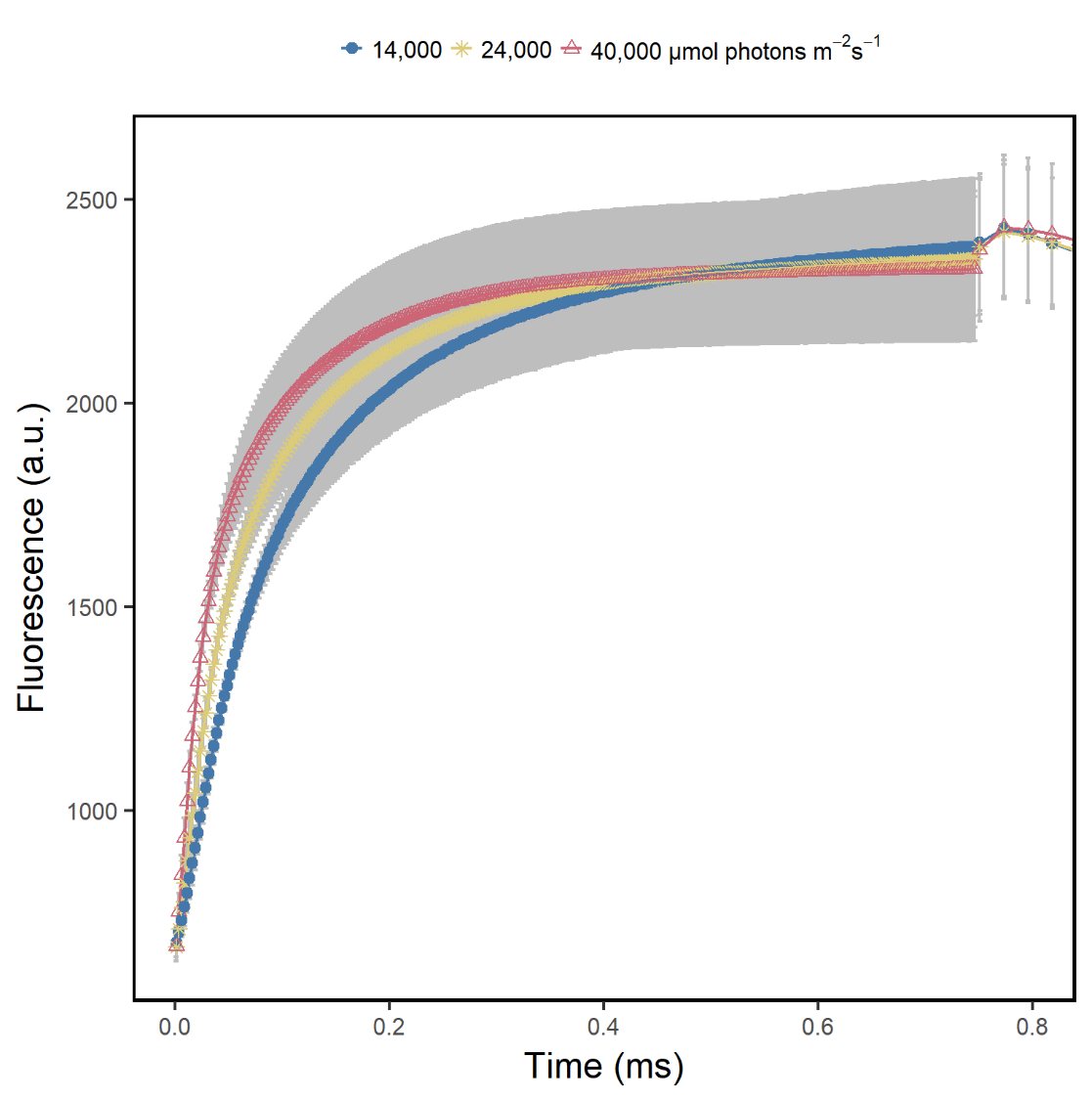


**Supplemental Figure S1.** Chlorophyll fluorescence transients measured in attached spinach leaves using fast repetition rate flash for 0.75 ms (FRRF_0.75ms_) under indicated excitation power. For comparison, chlorophyll fluorescence signal was normalized to excitation power. Plants were dark-adapted for 30 mins and measured from 0.6 m distance. Chlorophyll fluorescence transients are presented on a logarithmic time scale. Error bars show the standard error of the mean values (n=6 plants)
